# The Impact of Endocrine-Disrupting Chemicals in Male Fertility: Focus on the Action of Obesogens

**DOI:** 10.3390/jox11040012

**Published:** 2021-11-29

**Authors:** Luís Rato, Ana C. A. Sousa

**Affiliations:** 1 Health School of the Polytechnic Institute of Guarda, 6300-035 Guarda, Portugal; 2 Department of Biology, School of Science and Technology, University of Évora, 7006-554 Évora, Portugal; 3 Comprehensive Health Research Centre (CHRC), University of Évora, 7000-671 Évora, Portugal

**Keywords:** environmental contaminants, obesogens, infertility, reproductive axis, spermatogenesis, Sertoli cells, germ cells, sperm quality

## Abstract

The current scenario of male infertility is not yet fully elucidated; however, there is increasing evidence that it is associated with the widespread exposure to endocrine-disrupting chemicals (EDCs), and in particular to obesogens. These compounds interfere with hormones involved in the regulation of metabolism and are associated with weight gain, being also able to change the functioning of the male reproductive axis and, consequently, the testicular physiology and metabolism that are pivotal for spermatogenesis. The disruption of these tightly regulated metabolic pathways leads to adverse reproductive outcomes. The permanent exposure to obesogens has raised serious health concerns. Evidence suggests that obesogens are one of the leading causes of the marked decline of male fertility and key players in shaping the future health outcomes not only for those who are directly exposed but also for upcoming generations. In addition to the changes that lead to inefficient functioning of the male gametes, obesogens induce alterations that are “imprinted” on the genes of the male gametes, establishing a link between generations and contributing to the transmission of defects. Unveiling the molecular mechanisms by which obesogens induce toxicity that may end-up in epigenetic modifications is imperative. This review describes and discusses the suggested molecular targets and potential mechanisms for obesogenic–disrupting chemicals and the subsequent effects on male reproductive health.

## 1. Introduction

**Figure 1:**
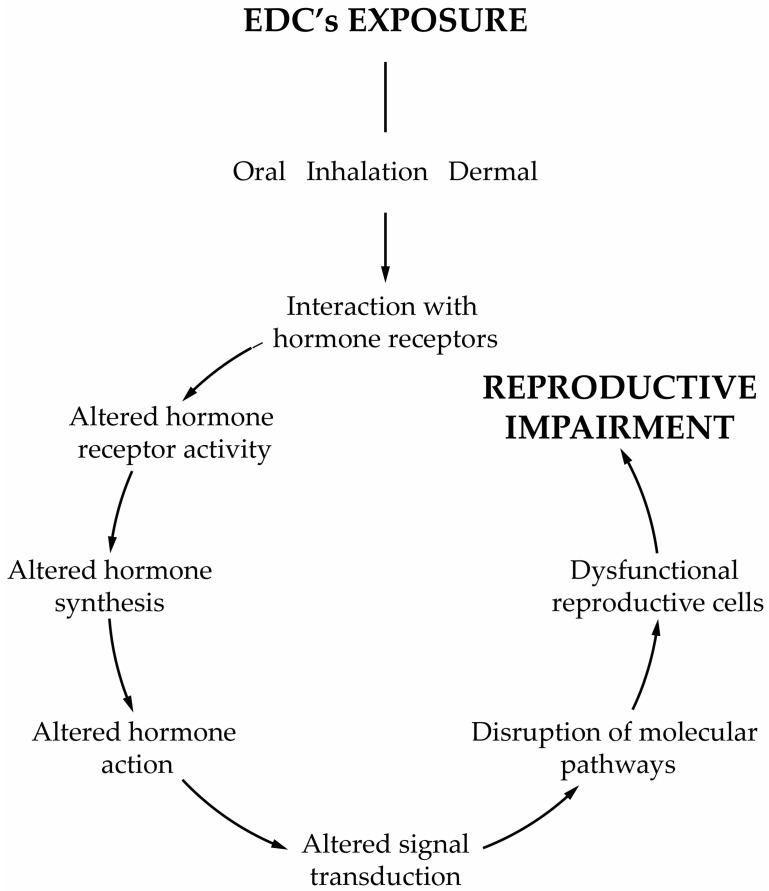
Schematic representation of the most important characteristics of obesogens’ effects.

Today, we are exposed to a cocktail of man-made chemicals, some of which have the potential to disrupt our hormonal system. Any compound or mixture of compounds, which interferes with any aspect of the endocrine system is classified by the Endocrine Society as an endocrine-disrupting chemical (EDC) [1]. These chemicals are ubiqutious in consumer products, being widely detected in the indoor environment (e.g., homes, workplaces, schools, gyms, transportation) and in diet samples. Generally, their occurrence in the indoor environment is a consequence of their widespread use in construction materials and consumer products, including, for example, house furnishings, electronic devices, wallpaper, textiles, kitchen utensils, cleaning products, and cosmetics, amongst many others. Indoor activities such as the use of biocides, deodorisers, the burning of incense and candles, smoking and cooking are also responsible for the release of EDCs into the indoor environment. Furthermore, all materials carried from outdoors on our shoes or clothes also increase our exposure. EDCs are also present in food items due to food production (e.g., pesticides), processing (antimicrobials, preservatives) and storage (plasticizers), which makes diet a significant route of EDCs. Such continuous and widespread exposure has raised concerns not only within the scientific community but also to the health authorities and policymakers due to the adverse effects on human health. As the endocrine system is at the interface with the environment, EDCs disturb the homeostatic mechanisms of the body. The endocrine system plays a central role in regulating critical biological functions as metabolism, development, reproduction, and behavior. Understanding how these chemicals affect physiologic processes and initiate pathological conditions is essential to dissect the etiology of hormone-related diseases. Scientific evidence has demonstrated the relationship between EDCs and neurobehavioral and neurodevelopmental changes, immune disorders, cancer development, reproductive health problems and metabolic diseases in humans [1,2,3]. All these findings are in line with what was reported from animal models [1,4,5]. The group of compounds classified as EDCs is extremely heterogeneous and includes industrially produced chemicals, such as polychlorinated biphenyls (PCBs), bisphenol A (BPA), phthalates, pesticides, fungicides, and pharmaceutical drugs [1]. Likewise, natural chemicals found in human and animal diets (e.g., phytoestrogens, such as genistein and daidzein, or even mycotoxins) can also act as EDCs [1]. Furthermore, several of these compounds are lipophilic which make them particularly relevant from a physiologic point of view. EDCs act by different mechanisms and elicit several responses. One recent concern about the adverse effects of EDCs is the ability that some of them have to induce weight gain [6]. Today these chemicals are classified as obesogens, a term coined by Blumberg and Grun in 2006 [7]. Obesogens are a limited group of EDCs capable of inducing adipogenesis and altering the mechanisms that regulate appetite, satiety and energy metabolism. Though the relationship between lifestyle habits of modern societies and the obesity rates has been established, obesogens have been considered major contributors to the obesity epidemic [8,9,10]. The mechanisms by which obesogens exert their effects are much broader than was initially postulated, see reviews [11,12,13,14,15]. In Europe, exposures to EDCs have contributed substantially to obesity [8,16,17,18,19] and albeit obesogens are only part of the problem, it is necessary to become aware of the risks of permanent exposure to these chemicals. Reducing our exposure to these chemicals is therefore important to decrease the chances of becoming more prone to develop obesity. In parallel to the adverse effects of obesogens on whole body metabolism, another issue of concern is male reproductive health problems resulting from exposure to EDCs, whether they are obesogenic or not. In the last decades, male fertility has decreased significantly. Today, 15% of the population is infertile, and one in seven couples is infertile at reproductive age [20]. About 50% of the cases are due to the male factor, and in many of them, it occurs due to idiopathic infertility [20]. The etiology of this adverse trend remains a subject of intense debate, but there has been a consensual awareness regarding the role of external factors, such as lifestyle habits and its associated factors, especially the permanent exposure to EDCs. The decline of male fertility has also been concurrent with the overall increase in exposure to EDCs, leading to these contaminants being singled out as an important cause of male infertility [21]. Treatment is usually associated with assisted reproductive technologies, which are applied to increase the chances of conception. Infertility is not a life-threatening disease, but it is vital to society, and the high costs associated with the infertility treatments and medically assisted reproduction techniques make these alternatives almost inaccessible to most infertile couples. The male reproductive function is highly susceptible to the effects of EDCs [21,22], and most of these chemicals are lipophilic, mimicking naturally occurring hormones. Male reproduction is highly dependent on endocrine regulation, especially the reproductive events, such as steroidogenesis and spermatogenesis that are dependent on Leydig (LCs) and Sertoli cells (SCs), respectively [23]. The majority of EDCs interfere with the receptors of endogenous hormones, impairing genome and non-genomic responses [24], which is extremely relevant since most of the effects are exerted through disturbance of estrogen-, anti-estrogen, androgen- and anti-androgen-mediated processes [25,26]. However, some EDCs can modify hormone bioavailability by interfering with its secretion and transport or by disrupting the enzymatic pathways involved in hormone synthesis and metabolism [27,28]. It is essential to understand how EDCs, especially obesogens, induce male infertility namely in the deregulation of the mechanisms that govern the function of the reproductive axis, sperm formation, and its functioning (Figure 1).

Information regarding human data is scarce due to ethical issues in performing human studies to unveil the adverse effects of EDCs. Furthermore, most of the available data demonstrated the effects of EDCs alone, and the understanding of the potential human health risks requires the study of the complex mixtures. In this context, animal studies have suggested that environmental cues play a significant role in sperm quality and thus, in male fertility [29,30,31]. However, there is a need to converge information from basic knowledge about EDCs’ mechanisms, and epidemiological studies to predict and assess potential effects in humans. Furthermore, EDCs can also act on multiple generations, through small modifications induced in the DNA of male gametes and contributing to a transgenerational amplification of reproductive defects, but also other adverse health outcomes.

**Figure 2:**
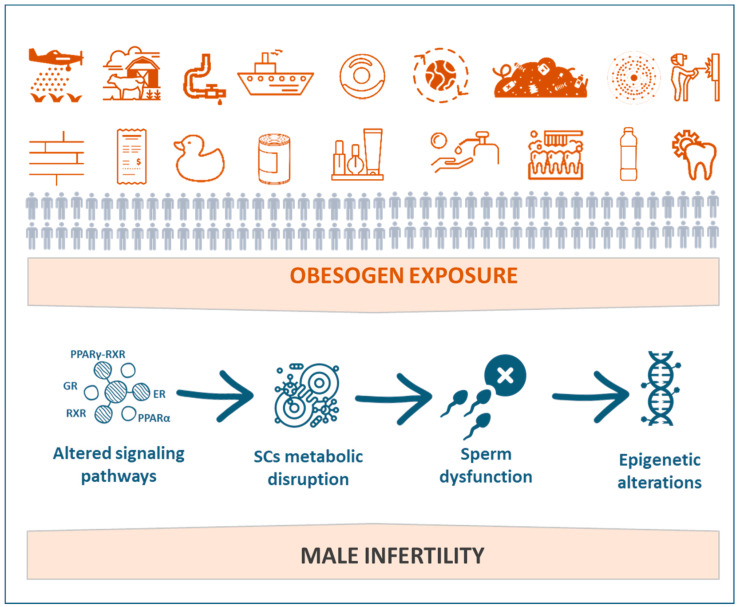
Schematic representation of the implications of exposure to obesogens on male reproductive impairment.

This chapter will present and discuss the impacts of EDCs, especially obesogens, on male reproductive health (Figure 2). The putative effects of obesogenic EDCs on sperm function and sperm-related events and the subsequent consequences to overall male fertility potential will also be explored.

## 2. Brief History of Endocrine-Disrupting Chemicals

In the beginning of the 20th century, pig farmers in the United States became concerned about the reproductive complications in their swine herds fed with a moldy grain [32]. Later in the 1940s, sheep farmers in Western Australia also observed reproductive problems in their sheep after grazing on specific fields of clover [33]. These were the first evidence of the effects of EDCs on animals. The idea that EDCs could find their way into the animals began to gain strength when Gassner and collaborators [34] described that the presence of certain chemicals in livestock feedlots could increase exposure. However, it was only in 1962, after the “Silent Spring” book, that the scientific community awakened to this issue. Rachel Carlson gave an important warning message of how the indiscriminate use of pesticides can leave “our fingerprint” in ecosystems, ultimately affecting ecosystems and human health. This wake-up call came to join the previous one by Bane-Jones, “The Advancement of Medical Research and Education through the Department of Health, Education and Welfare” [35] for the creation of an agency specifically committed to studying the effects of environmental exposure in human health. So, in 1966, the National Institute of Environmental Health Sciences (NIEHS) was created and four years later, together with the United States Environmental Protection Agency (US-EPA), they implemented regulations allowing the protection of the environment and human health. By that time, several studies provided clues on how chemicals caused endocrine disruption; for instance, evidence from minks showing reproductive complications and increased mortality rates, which were ascribed to fish from Great Lakes contaminated with PCBs [36,37]. By the same time, Gilbertson and Reynolds [38] observed low birth rates and high proportions of eggs failing to hatch in terns feeding from fish contaminated with hexachlorobenzene in the sea harbor. However, none could imagine that, around the 1970s, one of the most important medical breakthroughs would take place when the effects of diethylstilbestrol (DES) were discovered. DES is a synthetic estrogen, that had been used since 1930 for several indications, such as to reduce miscarriage or hormone therapy for menopausal women’s symptoms, or even estrogen deficiency. DES was later shown to be related to serious health consequences like rare cancers, reproductive malformations, and other conditions alerting the long-term effects of DES that were only noticeable after one or even more generations [39,40]. After that, EDCs’ research focused on the understanding of how certain chemicals accumulated and degraded in organisms [41]. The interest in EDCs was increasing, and the importance of their study was evidenced by the increasing number of scientific meetings dedicated to this topic. In 1991, one of the most important conferences, the Wingspread Conference, took place, where the concepts of “endocrine disruption” and “endocrine disruptors” were established [42]. From that date, the classical view of hormone function was revised, and it was apparent that there was more than one “key” for the same “lock”. The 1990s were prolific in demonstrating the harmful effects of EDCs. In 1992, Carlsen and collaborators [43] attracted the attention of the scientific community when they associated the falling sperm counts with the exposures to EDCs over the past five decades. In 1995, the US-EPA sponsored a workshop to gather interested parties with the aim to identify gaps regarding environmental influences on humans and wildlife. The workshop group felt the need to develop further investigations specifically regarding the health-effects of carcinogenesis, reproductive toxicity, neurotoxicity, immunotoxicity, risk assessment paradigm-hazard identification, dose-response assessment, exposure assessment, and risk characterization. In Europe, researchers assembled at the first European Workshop dedicated to the impact of EDCs, the Weybridge meeting [44], while similar events took place around the world [45]. In 1998, the US-EPA announced the “Endocrine Disruption Screening Program,” which was mandated under the Food Quality Protection Act and Safe Drinking Water Act to establish a framework for priority setting, screening, and testing of more than 85,000 chemicals in commerce. The idea behind the program was that prioritization would be based on existing information about chemical uses, production volume, structure-activity, and toxicity. Through the Registration, Evaluation, Authorization, and Restriction of Chemicals legislation, usually known as REACH, which became law in the European Union in 2007, chemicals, in which potential EDCs are included, require a portfolio of safety information before being released into the environment, rather than waiting for problems ahead, a fundamental aspect of the precautionary principle. With the growing scientific concerns and public debate about the potential adverse effects of EDCs exposure, the World Health Organization (WHO) published the global assessment of the state-of-the-science of EDCs with the purpose of providing an objective, global assessment of the current state-of-the-science relative to environmental endocrine disruption in humans, experimental studies, and wildlife species [46]. Eighteen years after Wingspread, the Endocrine Society of USA published a scientific statement showing how experimental and epidemiological data converge to implicate EDCs as one of the major concerns to human health [47]. In 2013, the WHO and the United Nations Environment Program released the most comprehensive study on EDCs [48], calling for more research to fully explore the association between EDCs and the risks to the health of human and animal life. In 2014, a multidisciplinary group of scientists, from basic to clinical settings, gathered in Parma for a workshop aiming to examine the relationship between EDC exposure and the increased incidence of metabolic disorders worldwide [49]. In 2015, the Endocrine Society of USA reviewed the latest science and updated the first statement on the recent knowledge and existing gaps [1]. There is a general and conclusive agreement about the EDCs’ hazards. The benefits of “chemical revolution” have shown us the “other side of the coin” where the subsequent environmental contamination is related to a wide range of behavioral, endocrine, and neurobiological complications. The research in this field has advanced rapidly; nevertheless, the strategy of health and politic authorities involved in the study of EDCs must be updated and revised in a continuum.

## 3. Endocrine Disruptors as Obesogens: Evidence from Basic and Clinical Studies

**Figure 3:**
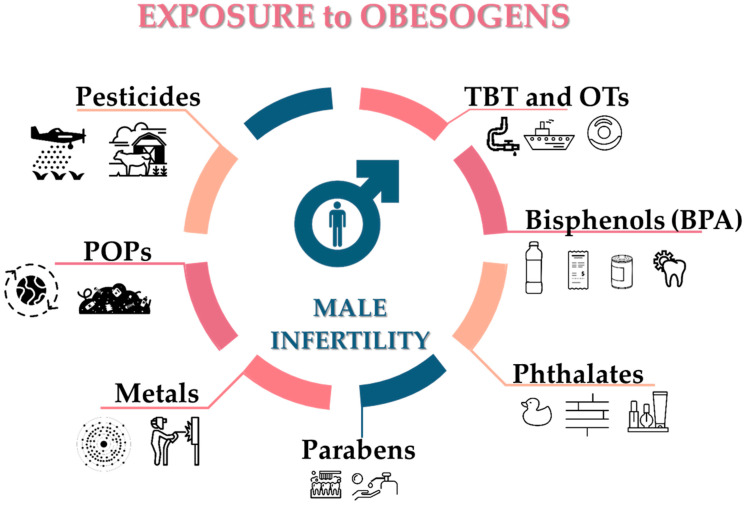
Potential sources of obesogens known to affect the male reproductive system. TBT: tributyltin; OTs: organotin compounds; BPA: bisphenol-A; POPs: persistent organic pollutants.

The endocrine system is known to play an essential role in energy balance, fat deposition, and fat distribution in the body because many endocrine organs and hormones work together to regulate metabolism and body weight. A significant advance in our understanding of how the environment can influence health, particularly by affecting developmental programming, came from the recognition that chemicals could disrupt the function of the endocrine system. However, the interaction of multiple factors across the lifespan and generations makes it difficult to determine what extent obesogenic EDCs contribute to the obesity pandemic in humans, despite its great importance and increasing interest from not only scientists, but also by health authorities, the general public and policy makers. Obesogens act as metabolic disrupters, promoting adipogenesis, increasing energy storage in fat tissue, and interfering with the hormonal control of appetite and satiety. There are nearly 50 known obesogens (Figure 3, Table 1), or chemicals supposed to exert obesogenic action (for review see [12,50]), and though some works are purely correlative [51], others have deepened understanding regarding the molecular mechanisms on how obesogens stimulate adipogenesis [52,53]. Obesogens interact with peroxisome proliferator-activated receptors (PPARs), particularly the peroxisome proliferator-activated receptor γ (PPARγ), the key regulator of lipid metabolism, adipocyte function and differentiation [54]. Studies have allowed us to understand the relationship between obesogens and weight gain better. One of the first studies demonstrated that tributyltin (TBT), a human-made chemical introduced into the environment mainly due to its widespread use as an antifouling agent in ships and other submerged structures, acts as a nanomolar-affinity ligand for PPARγ and its heterodimeric partner, 9-*cis* retinoic acid receptor (RXR) [7,20,55,56]. TBT is considered the obesogen model and belongs to the organotins which comprises of several families sharing structural similarities [57,58] (including e.g., butyltins, phenyltins and octyltins), being used in diverse applications including the aforementioned biocidal activities, in the production of polyurethane foams and silicones, and in the stabilization of plastics [59]. TBT is lipophilic so it may accumulate in our bodies [60,61,62,63,64,65]. It has been proposed that TBT commit cells to the adipocyte development by altering the chromatin landscape through activation of RXR, which decreases trimethylation in the lysine 27 of the histone 3 (H3K27me3), leading to increased expression of genes involved in adipogenesis [66]. Additionally, mesenchymal stem cells (MSCs) from mice exposed to TBT in the womb were biased towards the adipose lineage at the expense of the osteoblast lineage. This was confirmed under in vitro conditions where MSCs exposed to TBT were shunted toward the adipose fate in a PPARγ-dependent pathway. The activation of RXR seemed to be enough for adipogenic commitment, and the rexinoids acting through RXR activated it and altered the expression of the enhancer of zeste homolog 2, leading to H3K27me3, and thus promoting adipose commitment and programming subsequent differentiation [66]. Furthermore, in vitro studies have shown that TBT also increases basal glucose uptake in adipocytes exposed to 50 nM of TBT [67]. Similar to organotins, phthalates have also been classified as obesogens. Phthalates are esters of phthalic acid; high molecular weight phthalates are used mainly as plasticizers to increase the flexibility, transparency, and durability of plastic materials and can be found in several products of our daily life such as packaging, children’s toys, electronics, flooring and medical equipment. Low molecular weight phthalates are used as solvents in personal care products including cosmetics, air fresheners, food products, varnishes and coatings, pharmaceuticals and textiles. In vitro studies demonstrated that bis(2-ethylhexyl) phthalate (DEHP) and its metabolite mono-(2-ethylhexyl)-phthalate (MEHP) enhance differentiation of 3T3-L1 preadipocytes into adipocytes and murine MSCs in a dose-dependent manner through activation of the PPARγ pathway [68,69]. However, phthalates also stimulate adipogenesis by exerting antiandrogenic effects through PPARα. Another high production chemical widely used in polycarbonate plastics, can linings and thermal paper such as the one used in currency and in cash register receipts is bisphenol A (BPA), which had already revealed obesogenic action by predisposing murine preadipocyte differentiation, not only acting through nuclear estrogen receptors [70], but also via nongenomic mechanisms acting through cell membrane-associated estrogen receptors [71]. This is possible because BPA’s structure enables it to fit into the binding site of the estrogen receptor, which allows the compound to activate both nuclear and cell membrane-localized estrogen receptors [72]. This action directly modulates the insulin dependent phosphatidylinositol 3-kinase/protein kinase B (PI3K/AKT) pathway and regulates glucose uptake [73]. Other chemicals have been identified as potential obesogens by promoting adipogenic differentiation; for instance, parabens which are used as preservatives in personal care products, foods, pharmaceutical products, and paper products. The cell line 3T3-L1 exposed to 100 µM of ethyl- propyl-, and butylparaben demonstrated increased adipogenesis, showed lipid accumulation and an up-regulated activity of PPARγ [74], as well as an up-regulation in the transcription of PPARγ in C3H10T1/2 multipotent stem cells [75]. Moreover, it exhibited butyl- and benzylparaben-induced adipogenesis, though in a dose-dependent manner [76]. Parabens act as PPARγ agonists, but they can mediate their obesogenic effects through other pathways, such as the case of the glucocorticoid receptor [77] or through endocannabinoids as demonstrated by [76].

Several classes of persistent organic pollutants (POPs) were already associated with obesity development. Polychlorinated biphenyls (PCBs), for example, are POPs that were used in electrical and industrial equipment. PCB101, PCB153, and PCB180 impair leptin signaling in differentiated 3T3-L1 adipocytes with associated increased lipid content and reduction of the AMP-activated protein kinase/acetyl-CoA carboxylase pathway, which is directly involved in the fatty acid oxidation [78]. Organochlorine compounds (OCs), also POPs, and widely used as pesticides, for example, also alter increased basal free fatty acid uptake by mature 3T3-L1 adipocytes. This increase was followed by augmentation of adipokine release, namely adiponectin and resistin [79]. Other environmental contaminants, such as the metals lead, cadmium and mercury and some phytoestrogens (genistein and daidzein), also exhibit obesogenic effects. One should note that the effects vary with the time of the exposure, since EDCs present non-monotonic dose–response curves, with results for higher doses often being opposite from what was observed for lower doses. Genistein, for example, and in contrast to its effects in adults [80], may produce obesogenic effects in animals exposed at critical periods of development. Interestingly, mice exposed to lower doses of phytoestrogen during gestation and lactation present a lower birth weight, but develop obesity, high leptin, and 17β-estradiol (E_2_) levels, and impaired glucose metabolism [81]. In vitro studies have enlightened the molecular mechanisms by which obesogens enhance adipogenesis at environmentally relevant doses. Importantly, some of these studies have been performed using cell lines, and thus further studies to evaluate in detail the obesity risk assessment for humans from exposure to obesogens alone or in a mixture are required. Therefore, epidemiological surveys alongside animal models have a crucial role in this context, so their use must be heavily weighed in the assessment paradigms. Many obesogens alter the function of gene expression and the hormone secretion of the white adipose tissue, the number and volume of adipocytes, or the body weight of the animal after exposure. Zuo and collaborators [82] orally administered TBT (0.5, 5 and 50 µg/Kg) once every three days, and after 45 days of treatment, the authors observed a significant increase of the weight in the animals treated with the intermediate levels (5 µg/Kg) without changing food consumption. This was concurrent with an altered hormonal profile of metabolic hormones, where the levels of leptin and resistin were significantly increased, while the release of adiponectin was decreased. Increased levels of leptin and resistin are linked with obesity [83,84], and this may result from mechanisms such as those previously described [66]. Nevertheless, it is possible that TBT may exert estrogenic activity as reported by Penza and collaborators [85]. In another study where pregnant rats were dosed daily by gavage with chlorpyrifos (2.5 mg/Kg) from gestational day seven until post-natal day 21 (PND21) (end of lactation), an abnormal weight gain in the offspring was observed. Similarly, Vitalone and collaborators [86] found that pregnant rats, after being exposed from gestational day 7 to PND21 to low doses of methylmercury (0.5 mg/Kg/day in drinking water) and PCB126 (100 ng/Kg/day in food), exhibited increased weight gain in the males of the offspring. This not only illustrates the obesogenicity of such contaminants, but also elicits a gender-specific development of obesity. The obesogenic action of BPA in male rats perinatally exposed has also been described, which exhibited overweight and significant fat accumulation.

All this crucial evidence demonstrates how obesogens act in a specific model, whether cellular or animal; however, it must be taken into account that in some instances the mechanisms may be merely indicatively associated with specific endpoints (weight, fat accumulation, hormones levels, and others). The association between EDCs and health outcomes in humans is nevertheless even more difficult to prove. Most of the unequivocal evidences available so far are derived from acute high dose exposures that occurred throughout history due to regrettable accidents [105,106]. The establishment of cause-and-effect links between exposure and diseases comes from examples such as industrial disasters with dioxins in Seveso, Italy and in Yusho, Japan, which resulted in severe births defects and neurocognitive impairments in children. Those were moments of acute exposure to high levels of EDCs, but demonstrates the adverse health outcomes [105,106]. However, the exposure to EDCs occurs at low doses in a chronic way, throughout our lives, from pre-conception until late age. Thus, such continuous exposure to a myriad of chemicals renders the establishment of a causation almost impossible, nevertheless several epidemiological evidences strongly suggest associations between exposure to EDCs and obesity. Carwille and Michels, for example, found an association between urinary BPA levels and obesity when analyzing data from the 2003–2006 National Health and Nutrition Examination Survey (NHANES) [107]. Similarly, Shankar and collaborators [108] found a positive association among urinary BPA levels, body mass index and waist circumference based on data from 2003–2008 NHANES. Indeed, these studies have suggested that obesogens are contributing to obesity epidemics, regardless of poor nutritional diet content or physical activity. In a longitudinal observational study, exposure to POPs was associated with rapid growth and with being overweight [109]. This is consistent with what was found by Mendez and collaborators [110], who explored the effects of prenatal exposure to organochlorine compounds and found an association with rapid growth in the first months of life and increased BMI later in the infancy. In a Swedish cohort of individuals aged > 70 years, it was demonstrated that serum levels of mono-isobutyl phthalate were associated with BMI, waist circumference and total fat mass [111]. Recently, Tang-Peronard and collaborators [112] observed that prenatal exposure to PCBs and p,p’-dichlorodiphenyldichloroethylene (DDE) might play a role for subsequent obesity development in infancy, with the most pronounced effects being associated with the exposure to low doses. The pharmacological route can also be pointed out regarding the exposure to obesogens, for example, through the use of antidiabetic drugs. Thiazolidinediones belong to the class of oral antidiabetic drugs that improve insulin sensitivity. These drugs exert their antidiabetic effects through a mechanism involving the activation of PPARγ and had been already associated with persistent weight gain [113,114]. Although the epidemiological evidence demonstrates an association between exposure and health outcomes, it is essential to bear in mind that most of these works lack mechanistic explanation due to the limitations of these studies. Most of them fail to consider the exposure levels relative to critical exposure windows. For some studies, there is no consideration regarding confounding factors, such as diet, stress, and others, as well as the latency of obesogens effects. These are vital points that must be taken into account in the design of future studies. Besides, it is also necessary to consider that some obesogens present short-half lives and that they may be converted into their metabolites, so researchers conducting studies on the effects of certain obesogens may not evaluate the real effects of the former. Furthermore, obesogens exert their action through several pathways [115], and most of the human studies were not designed for such an elaborated framework.

## 4. Obesogens as a Threat to Male Fertility

The obesogen hypothesis proposes that exposure to obesogens affects the biochemical pathways that control the whole metabolic homeostasis. Obesogens are present everywhere, and one of the first studies demonstrating that obesogens could affect the reproductive capacity through “modulation” of the endocrine system dates from the late 1960s [116]. Since then, several reports evidenced that the primary concern regarding obesogens is based on their capacity to induce endocrine disruption [1]. Taking into account that today the time period in which men are exposed to obesogens is large, this issue deserves special attention from all professionals of the reproductive area to understand how these toxics impact male fertility. Compelling evidences have linked the decline of male reproductive health with permanent exposure to obesogens in both developed and developing countries [117,118]. Obesogens alter the male reproductive function primarily by exerting adverse effects on the central nervous system. Below, a brief description of the effects of obesogens in the neuroendocrine control of male fertility, namely in the hypothalamus, is provided. Male reproductive organs may be a free spot where obesogens can be stored, albeit this is a subject of conjecture [119]. It is known that obesogens effectively cross the biological barriers [120], but the extent to which environmental chemicals pass the blood–testis barrier (BTB) remains mostly unknown. There is a correlation between obesogen exposure and decreased testosterone (T) levels, as they modulate the steroidogenic function of LCs (Table 2) and all related events with the synthesis of sex steroids [121]. Furthermore, studies also proposed that obesogens affect testicular metabolism, which is highly dependent on glucose [122,123,124]. Here we will explore how the metabolic cooperation between SCs and germ cells is affected by obesogens (Table 2) and possible consequences to spermatogenesis, and therefore male fertility. The effects of obesogens in the germ cells and sperm quality will also be examined.

## 5. Reproductive Axis at the Interface with Environmental Obesogens

The central neuroendocrine system controls all processes in the body, reproduction included. All molecular events are initiated at the base of the brain, where the hypothalamus is located, which serves as the primary interface between the central nervous system and the testicles, in this case. The hypothalamic neural cells synthesize and release the gonadotropin-releasing hormone (GnRH) into the capillary portal system that vascularizes the anterior pituitary. The gonadotrophs in the pituitary response to this stimulus is the release of the corresponding hormones, luteinizing hormone (LH) and follicle-stimulating hormone (FSH) into the bloodstream. FSH and LH act as a functional link between the brain and testes by working on testicular cells to regulate the spermatogenic event [134]. LH binds to membrane receptors of LCs and stimulates T synthesis, influencing the development of peritubular cells, SCs and, consequently, germ cells [135]. LCs irreversibly convert T into E_2_ by aromatase P450 [136]. On the other hand, FSH binds to membrane receptors on SCs, stimulating the production of E_2_, activin and inhibin B. E_2_ inhibits T production by LCs, while activin and inhibin B produce a positive/negative feedback on the pituitary, respectively. There is evidence that endocrine disruptors affect the neuroendocrine systems, and most of the studies have focused on the hypothalamus–pituitary–testicular (HPT) axis, termed the reproductive axis. Endocrine disruptors can exert diverse actions over the target cells according to their chemical structure and activities. In vitro evidences demonstrate that GnRH GT1–7 cell lines exposed to low doses of PCB mixtures, Aroclor 1221 or Aroclor 1254, increased the expression of the GnRH gene [137]. In this case, the endocrine disruption occurs at the level of the hypothalamus [137]. Similar studies using GT1–7 cell lines exposed to small doses of organochlorine pesticides demonstrated an increased expression of GnRH, whereas when exposed to high doses, the opposite effect was observed [138]. Although these works showed a positive response of GnRH cell lines to environmental obesogens, other studies demonstrated different outcomes. In fact, explanted hypothalamus from male rats exposed in utero to dioxins displayed an increased content of GnRH peptide, which was, however, accompanied by an impaired release [139]. Obesogens can favor or reduce the release of neuroendocrine hormones, but this will depend on the type of obesogens, the time of exposure and perhaps the levels to which disruption in the reproductive axis occurs. Different environmental compounds may target the HPT axis at various sites with different intensities, disrupting its regulation. BPA is an example of an obesogen acting through a variety of physiological receptors. BPA molecules are linked by ester bonds that are subject to hydrolysis when exposed to high temperatures or too acidic or basic substances [140]. BPA was thought to exhibit a weak estrogenic activity, based on the relative binding affinity of BPA for the nuclear estrogen receptors (ERs) α and β, which were estimated to be over 1000–10,000-fold lower when compared to E_2_ [141]. However, BPA binds to both estrogen receptors α and β [142,143] and plasma membrane-bound ERs [144], as well as to the G-protein-coupled receptor 30, showing that beyond the genomic route, BPA also act by non-genomic pathways. Rodents exposed to BPA during the perinatal and postnatal periods exhibited an upregulation of kisspeptin (KiSS-1) expression levels, GnRH and mRNA of FSH [145]. KiSS-1 acts as a guardian to the onset of puberty and for the regulation of gene expression in the HPT axis. The upregulation of KiSS-1 expression stimulated the synthesis and release of GnRH and gonadotropins in the hypothalamus and pituitary, respectively [145]. However, it was later shown that the hypothalamus is not involved in the disruption caused on the HPT axis by an adult exposure to BPA [146]. In this study, the relative transcript levels of the GnRH receptor, the gene which encodes the receptor for type 1 GnRH, was increased by five-fold in the pituitary of BPA-exposed groups [146]. Since GnRH is responsible for the release of FSH and LH from the anterior pituitary, as expected, both relative levels of LHb mRNA and FSHb mRNA, the corresponding genes, were also increased. Although the increased GnRH receptor expression may correspond to augmented T levels, this increase in androgen and E_2_ receptors suggests the stimulation of negative feedback mechanisms [147]. The same compound was also related to an imbalance in the ratio T/E_2_ due to an enhanced aromatase activity that increases E_2_ levels [148]. As a result, the release of gonadotropins at the hypothalamic-pituitary axis is inhibited, compromising all downstream events of the HPT axis, namely spermatogenesis. TBT is also involved in the dysregulation of the HPT axis by inducing leptin resistance and increasing plasma insulin levels, accounting for reduced gonadotropin secretion at the hypothalamic-pituitary level [82]. Phthalates may promote insulin resistance, considering reduced gonadotropin secretion at the hypothalamic-pituitary level [149]. Insulin may directly act through its receptor located in the hypothalamus and pituitary [150], being pivotal for a normal function of the HPT axis [151]. From the perspective of potential reproductive onset, puberty can be defined as activation of the HPT axis, leading to steroidogenesis, gametogenesis and the development of secondary sexual characteristics. Thus, many aspects resulting from the functioning of this complex system are also susceptible to the exposure of environmental contaminants and modulation of the HPT axis.

## 6. Leydig Cells Are a Sensitive Target of Obesogens

The synthesis of sex steroid hormones occurs in the Leydig cells (LCs) which are extremely sensitive to the toxic effects of environmental contaminants. The deleterious effects of EDCs often conduct the impairment of hormone synthesis since they are associated with the inhibition of the activities of enzymes involved in steroidogenesis. Lipids are essential for this process and cholesterol is one of the primary precursors, so it would be expected that any dysregulation in the homeostasis of these components compromises the synthesis of steroid hormones. Indeed, the pesticide 2,4-dichlorophenoxyacetic acid (2,4-D) decreases the expression of 3-hydroxy-3-methylglutaryl coenzyme A synthase (HMG-CoA synthase) and reductase (HMG-CoA reductase) in LCs of mice [152]. These enzymes are pivotal for cholesterol synthesis, where HMG-CoA synthase is responsible for the condensation of acetyl-CoA with acetoacetyl-CoA to form HMG-CoA, which is reduced to mevalonate for the synthesis of cholesterol. This is one of the most important steps of the cholesterol biosynthesis, where the enzyme responsible for this committing conversion is HMG-CoA reductase. The impairment of cholesterol synthesis after exposure to 2,4-D disturbs testosterone synthesis. In the steroidogenesis, cholesterol is then carried from the outer mitochondrial membrane by steroid acute regulatory protein (StAR), where it is converted to pregnenolone via P450 side chain cleavage enzyme (P450scc). Then, this intermediate proceeds to the smooth endoplasmic reticulum where 3β-hydroxysteroid dehydrogenase (3β-HSD) turns it into progesterone. Progesterone is converted by P450c17 into 17-hydroxyprogesterone and into androstenedione, where the enzyme 17β-hydroxysteroid dehydrogenase converts it into T. It has been demonstrated that exposure to plasticizers such as di(n-butyl)phthalate decreased the expression of cholesterol transport genes such as StAR, the high-density lipoprotein receptor, also known as SRB1 [153]. Furthermore, the expression of genes involved in T biosynthesis, namely P450scc, 3β-HSD, and P450c17, was downregulated, which probably contributes to T deficiency [154]. It seems that the plasticizer DEHP exerts anti-androgenic effects directly onto LCs, inhibiting T synthesis probably through dysfunction of CYP17 [155]. However, the impact of its metabolite, MEHP, is much more pronounced since it inhibited the expression of all steroidogenic enzymes, as well as all T precursors, in both the Δ4 and Δ5 steroidogenic pathways [155]. The effects of both phthalates appeared to be specific for steroidogenesis since they did not alter the expression of the insulin-like 3 gene, a specific marker of LCs. In vivo models have also provided evidence of the cytotoxic effects of similar obesogens in testicular function as observed after daily exposure to 200 mg/kg/day of BPA for six weeks. Such exposure not only revealed the same results, but also disclosed a reduced number of LCs [156]. Similarly, Jin and collaborators [157] reported that after oral administration of 2 μg/kg/day of BPA during 14 days, male rats showed T deficiency due to decreased expression of steroidogenic enzymes. An in utero exposure to linuron, a urea-based herbicide, was revealed to affect fetal testis gene expression and to reduce T production in adult male rat offspring [158]. Curiously, unlike phthalate esters that reduce T production, linuron did not affect the expression of steroidogenic genes [158]. Linuron seems to bind to the androgen receptor but, interestingly, the set of malformations displayed after an in utero exposure to this compound [159] differs from other androgen receptor antagonists [160]. Thus, it is suggested that decreased T production induced by linorun may be due to an alternative mechanism in the absence of cytotoxicity. Linuron is also causally related to changes in sex differentiation of the male reproductive tract [161], which allows hypothesizing that the inhibition of T synthesis along with androgen receptor antagonism may contribute to impact the androgen signaling pathway. Alterations in sex differentiation were also found after exposure to vinclozolin, a fungicide with anti-androgenic properties as well [162]. Indeed, the female-like anogenital distance at birth, retained nipples, undescended testes, and small sex accessory glands, were associated with an in utero vinclozolin exposure in male rat offspring [162]. The anti-androgen effects of vinclozolin were already related to increased levels of LH and T [163], and also inhibited DHT-induced transcriptional activation, through blockage of the androgen receptor-binding to androgen response elements in DNA [164].

## 7. Mechanisms Mediating Obesogen-Related Sertoli Cell Dysfunction: A Metabolic Standpoint

Typically known as “nurse cells”, Sertoli cells (SCs) are extremely important in male fertility, ensuring all physical and nutritional support of the germ cell line. Spermatogenesis is under the strict control of hormonal and endogenous factors [165,166], so SCs become an easy target of obesogenic EDCs mimicking endogenous hormones [167]. EDCs (obesogens included), not only compromise all structure of SCs, but also induce various cellular and molecular insults [168,169]. Additionally, the interactions between adjacent SCs are disrupted, provoking the premature exfoliation of germ cells [170]. The impact of EDCs on the integrity of cytoskeleton disruption of SCs and BTB has been extensively explored and well documented by Cheng and his collaborators [22,120,169,171,172,173,174,175,176]. Here, we will focus on the significant findings concerning the effects of EDCs, particularly obesogens, in the modulation of SC’s metabolism, since it is vital for the control of spermatogenesis [165,177,178] and because several EDCs have already been reported to alter factors that control the metabolism of these cells (Table 2). During the 1980s, researchers focused in demonstrating how environmental contaminants modulate the metabolism of SCs. Batarseh and collaborators exposed SCs under ex vivo conditions to 10 µM, 50 µM, and 100 µM lead acetate and observed a significant production of lactate in all concentrations tested [127]. SCs increased lactate production in a dose- and time-dependent manner after being exposed to lead acetate [127]. Similarly, a dose-dependent increase in lactate secretion was also reported in primary cultures of SCs exposed to other contaminants with recognized obesogenic activity, such as phthalate esters [124] and PCBs [128]. Increased lactate levels induced by these obesogens are often viewed as evidence of enhanced lactic fermentation. Those reports demonstrated that the quantification of the lactate released from cultured SCs can be used as a measure of the increased glycolytic pathway. Although these works were based on a basic approach, they provided unequivocal proof that EDCs may reprogram the metabolism of SCs with possible adverse consequences on male reproduction. However, the information that existed to date did not allow mechanistic perception of the toxicants’ performance. A few years ago, Premendu P. Mathur and his team shed light on how some obesogens compromise glucose metabolism. Their work showed that BPA disrupts the glycolytic metabolism of SCs by blocking the glucose movement through binding to the glucose transporters in the pore region [123]. BPA also disrupts testicular insulin signaling, by decreasing the expression of insulin receptor substrate 1, insulin receptor substrate 2, and PI3K [123]. PI3K is involved in the control of glucose uptake, namely in the translocation of glucose transporter isoform 1 (GLUT1) to the cell surface [179], so this leads to significant alterations on the glycolytic pathway. Under normal conditions, glucose is taken up by SCs through GLUT1, glucose transporter isoform 2, or glucose transporter isoform 3 (GLUT3), and readily converted to pyruvate through the glycolytic pathway, where phosphofructokinase 1 (PFK1) is the first rate-limiting step [180]. The resulting pyruvate is mostly converted to lactate by the action of lactate dehydrogenase (LDH), with a part being also converted to alanine. Previous reports showed that plasmatic concentrations detected in men exposed to 2,4-D are enough to decrease not only the intracellular levels of glucose, but also the transcript levels of glucose transporter 3 (GLUT3), PFK1 and LDH [122]. Similarly, lactate, alanine, and monocarboxylate transporter isoform 4 (MCT4), the carrier through which lactate is exported to the luminal fluid to be used as a metabolic fuel by the developing germ cells, were decreased as well. Besides, to compromise the intracellular levels of lactate, the exposure to these doses of 2,4-D also reduced the lactate/alanine ratio, which is an indicator of a reduced cytosolic state, and thus hampers the glycolytic metabolism of rat SCs [122,181]. The decrease on PI3K also downregulates the stimulation of lactate production and LDH activity, illustrating the impact of the PI3K/AKT signaling pathway in SCs metabolism [179]. Besides, these changes are also closely related to the apoptotic process [182], suggesting that these metabolic alterations may lead to abnormal apoptosis in SCs and thus induce problems on spermatogenesis and male fertility. It is known that model obesogen TBT alters glucose consumption mainly through disruption of the translocation mechanism mediated by GLUT1 [183]. Cardoso et al. [132] showed that SCs exposed to nanomolar concentrations of TBT exhibit alterations in the glycolytic profile. Ex vivo cultured rat SCs were exposed to 10 nM of TBT for 6 h and exhibited a significant decrease in glucose consumption even though the expression of GLUTs was unaltered. Moreover, these cells also decreased the consumption of pyruvate. Indeed, TBT at nanomolar concentrations reduced the consumption of the two significant substrates used to ensure lactate production. Hence, the striking decrease in the consumption of these two substrates was reflected in a substantial reduction in the production of lactate in SCs exposed to 10 nM of TBT. However, the most remarkable effects were observed when SCs were exposed to subnanomolar concentrations TBT (10^−2^ nM). In this group, SCs exhibited a different metabolic response since the decreased glucose consumption was concurrent with a down-regulation of the expression of GLUT1, and the production of lactate was lower than SCs exposed to 10 nM TBT groups [132]. TBT inhibits glucose uptake by primarily affecting the translocation of GLUT1 to the plasma membrane. The reduced levels of GLUT1 may be associated with the decreased activity of 5′ adenosine monophosphate-activated protein kinase (AMPK), which is involved in the translocation of GLUT1 for membrane surface, thus enhancing glucose uptake [184]. Pioglitazone is an antidiabetic drug, and similar to the TBT, is a potent PPARγ agonist [54]. Pioglitazone exerts positive effects in male reproductive health, and it has been demonstrated that pioglitazone modulates the glycolytic metabolism of human SCs [104]. Human SCs exposed to 1 and 10 µM of pioglitazone did not alter glucose uptake, but when exposed to pharmacological concentrations (100 µM) significantly increased glucose uptake via GLUTs without changing their protein levels [104]. SCs exposed to pioglitazone 100 µM increased the efficiency of the glycolytic flux and lactate production since the lactate production doubled and was positively correlated with key intervenients of glycolysis, such as MCT4 protein levels [104].

## 8. How Can Germ Cells Be Affected by Obesogens?

Compelling evidence shows that environmental contaminants induce several dysfunctions associated with the testicular dysgenesis syndrome, including seminiferous tubules atrophy and germ cell degeneration [185]. Furthermore, lipids are essential components of the membranes of germ cells. Deficient lipid incorporation into these cells leads to a defective germ cell structure and contributes to the impairment of sperm parameters, and consequently to alterations of sperm functionality. The oral administration of TBT has also been associated with induced apoptosis in testicular germ cells in prepubertal mice [119]. Those authors did not describe the molecular mechanisms by which germ cell apoptosis was induced, but according to recent evidence, it seems that environmental contaminants activate both intrinsic and extrinsic pathways of apoptosis. Mitra and collaborators [133] observed a caspase-3 activation and increased levels of pathways of phosphorylated c-Jun N-terminal kinase (JNK) and mitogen-activated protein kinase p-38 (p38-MAPK) in SC–germ cell co-cultures after being exposed to 600 nM of TBT. JNK activation serves as a pro-apoptotic signal and regulates the mitochondrial apoptotic pathway through a balancing act between the activation of pro-apoptotic members and inhibition of anti-apoptotic members of the Bcl2-related protein family [186]. On the other hand, the p-38 caspase-independent pathway seems to be also activated by TBT and thus plays a role in the germ cells’ death [187]. Other studies have also associated TBT exposure to the induction of apoptosis in testicular germ cells, since more apoptotic cells were found in the seminiferous tubules of 21-day mice after oral administration of 25, 50 or 100 mg of TBT/kg/day, for three days [188]. Germ cells are also targeted by DES since apoptosis of spermatogenic cells was observed in prenatal and neonatal mice exposed to this compound from gestational day 12 to PND20 [189]. In healthy individuals, germ cells present high levels of polyunsaturated fatty acids (PUFAs), which are crucial to their membrane fluidity and flexibility, and thus for fertilization. Since these cells are, however, unable to synthesize PUFAs, they take these fatty acids from SCs and incorporate them into phospholipids, via lysophosphatidic acid acyltransferase 3 [190]. Several studies already suggested that the current dietary habits, particularly the excessive consumption of high-energy diets, with high content in saturated fats and trans fatty acids, decreases the activity of Δ5 and Δ6 desaturases [191]. It was proposed that the limitation of PUFAs incorporation in membranes of germ cells adversely affects testicular lipid metabolism and thus contributes to the impaired production of sperm [192].

## 9. Epigenetic Changes in Germ Cells

The permanent exposure to environmental chemicals may induce epigenetic modifications. These modifications do not alter the DNA sequence and mostly occur through DNA methylation, histone, and long non-coding RNAs modifications, remodeling of nucleosomes and higher order chromatin reorganization, thus defining gene expression [193]. Germ cells are the “vehicles” in the transmission of genetic information to the next generation. Epigenetics also ensure that daughter cells have the same phenotype as the parental cell, controlling gene function from one generation to the next [193]. Indeed, the role of epigenetics varies between somatic and germ cells, as the latter undergo meiosis, which is of particular importance to maintain genomic integrity [194]. The epigenetic programming of the germline begins during the migration of primordial germ cells in the embryo [195]. Upon initiation of determination of gonadal sex, the primordial germ cells remethylate DNA and develop the germ cell lineage in a gender-specific manner [195]. Between the 12th and 15th days of embryonic development [196], the fetal testes start expressing steroid receptors, thus becoming more sensitive to the adverse effects of EDCs [197]. So, EDCs acting up during gonadal sex determination could reprogram the germ line through DNA methylation [5]. Because of the elapsed time between exposure and the detectable impacts in offspring (mainly at their adulthood), animal models have been beneficial to study those mechanisms. Data from animal studies supported that such exposure may affect the male reproductive function of offspring, determining the success of fertility. It has been shown that exposure to vinclozolin or methoxychlor during the remethylation programming of the germ line in rats is associated with induced aberrant sexual differentiation, gonad formation, and reproductive functions of the following generation [198,199]. Similar findings were found until the fourth generation, with an increased spermatogenic cell apoptosis and reduced number and motility of sperm being observed [196]. This transmission of the exposure-induced phenotype in a transgenerational manner suggests an epigenetic alteration in the DNA methylation pattern of the male germ line, namely an epigenetic mechanism involving reprogramming of the germ line. Possibly through abnormal regulation of DNA (cytosine-5)-methyltransferase 3 alpha and DNA (cytosine-5)-methyltransferase 3-like, methyltransferases are required for methylation of most imprinted *loci* in germ cells [200]. It seems that these changes are implicated in the transgenerational effects aforementioned. Thus, male fertility problems were suggested to be caused by a change in genome-wide methylation and, consequently, in gene expression. Dolinoy and collaborators also confirmed the adverse effects in males that were not directly exposed to BPA. The authors showed that early developmental exposure to BPA can change offspring phenotype by stably altering the epigenome, an effect that can be counteracted by maternal dietary supplements [201]. Interestingly, DNA methylation can be counteracted by maternal dietary supplementation with a source of methyl groups such as the phytoestrogen genistein, which has been considered an endocrine disruptor [201]. Regarding phthalates, namely DEHP, it was reported that when given to outbred mice between days 7–14 of embryonic development, it reduced both counts and motility of sperm in male offspring beyond the F3 generation [202]. Accordingly, it was suggested that DNA methylation was the potential epigenetic mechanism underlying these alterations since 16 differentially methylated and expressed genes were identified in newborn pups. Thus, this group of genes is suggested to hold clues on how DEHP acts transgenerationally. Studies in humans were already performed in order to understand the environmental influence in epigenetics. DES, which has been associated decades ago to the presence of hypospadias in human males exposed in utero [203] or whose mothers had been exposed in utero [204], was also reported among unexposed grandsons of women who had been exposed in utero [205]. The exposure occurred during the development of the reproductive system in primordial germ cells of the fetuses, being subsequently transmitted across generations to affected sons and grandsons [205]; although the underlying mechanism is not entirely explained, it may rely on epigenetic changes in the androgen receptor. Modifications in the histones also play a crucial role in epigenetics, since the placement of histone modifications in sperm contributes to the establishment of embryo constitutive heterochromatin [206]. Histone modifications have been shown to be pivotal in changing chromatin structure and, therefore, DNA accessibility [207], being able to regulate gene expression, chromatin remodeling, cell survival and cell death [207]. Histone modifications comprise methylation, acetylation, phosphorylation, ubiquitination, ribosylation, and sumoylation, which can be easily induced and removed by a wide range of enzymes [207]. The functional effects of these modifications depend both on the modified amino acid and the specific covalently attached group [208]. Acetylation is involved in direct histone assembly and helps regulate the unfolding and activation of gene expression, whereas methylation restricts the access to the transcription machinery to DNA [208]. It seems that valproic acid, a commonly prescribed anticonvulsant and mood stabilizer [209], and methoxyacetic acid, a solvent usually used in paints, dyes and fuel additives, can inhibit histone deacetylases exerting broad effects on DNA [210]. Additionally, paternal dietary folate seems also to improve offspring’s susceptibility to congenital disabilities through changes in histone methylation [211]. Interestingly, it has been observed that fathers fed with a high-fat diet evidenced changes in sperm histone composition, although DNA methylation of several imprinted *loci* appears to remain static [212]. These findings illustrate that men exposed to these contaminants are more predisposed to experience histone modifications. Long non-coding RNAs (lncRNAs) modifications are another epigenetic-related mechanism that can be pointed out as an effect of exposure to environmental contaminants. The role of lncRNAs in epigenetic processes has been highlighted, and several data have suggested that lncRNAs are involved in the cellular stress response [213]. LncRNAs are defined as transcribed RNA molecules greater than 200 nucleotides in length with little or no protein-coding competence. It is known that lncRNAs regulate gene expression, although the mechanisms need to be clarified in-depth [214,215]. It has been described how lncRNAs control gene regulation at every level including transcriptional gene silencing via DNA methylation and regulation of the chromatin structure [215,216]. It is likely that lncRNAs drive significant exposure–disease associations and may also function as biomarkers of environmental exposure. Studies have reported an increased transcription of telomeric repeat-containing RNA and satellite sequences (Sat III) in humans, following physiological stress induced by heat or ethanol [217,218]. This may indicate that lncRNAs have a transcriptional regulatory role, as suggested for other similar RNAs such as telomeric repeat-containing RNA and Sat III [217,218]. Likewise, satellite DNA transcription also appears to be regulated by environmental cues such as temperature [219]. Similarly to lncRNAs, small non-coding RNAs (sncRNAs) seem to be involved in mechanisms of histone modifications, specifically piwi-interacting RNAs (piRNAs). In fact, it has been observed that lncRNAs and piRNAs in the landscape of epigenetic modifications of chromatin state and histone codes are the productions of piRNAs by the lncRNAs that induce up-regulation of the tumor necrosis factor related to apoptosis-inducing ligand protein via H3K4/H3K27 methylation/demethylation [220]. It seems that these RNAs may be important mediators in the response to a broader spectrum of contaminants, and reproductive biologists are searching for further understanding of their biological functions. This is pivotal to identify the functional role of these RNAs in the toxic response of mammalian cells. There is consistent data which links epigenetic changes both in experimental and epidemiological studies to environmental contamination and subsequent effects in sperm quality, but there is a lot of work to do in this topic. Because these epigenetic changes are small, potentially cumulative, and they may develop over time, it may be difficult to establish real cause–effect relationships among environmental contaminants, epigenetic changes, and diseases etiology. However, knowing the relevance of the transgenerational epigenetic inheritance to many human disorders will undoubtedly become an exciting area, for which research on germ cells is particularly important.

## 10. Obesogens Compromise Sperm Quality and Sperm Function-Related Events

Spermatogenesis is extremely sensitive to the effects of EDCs. Any damage in this event will compromise germ cell differentiation into fully developed sperm. In this section, the adverse effects of EDCs on sperm and the putative impact ECDs on the molecular events associated with their function since their release from seminiferous tubules will be addressed. EDCs interfere with several aspects of sperm function and induce effects that, although are not so evident as the conventional parameters used by clinicians, should be considered as specific biomarkers of exposure. These biomarkers include, for example, oxidative stress induction, DNA damage, fluctuations in the intracellular calcium concentration [Ca^2+^]_i_ and mitochondrial dysfunction. There are several studies on this topic, however with inconsistent results (for more details see Table 3 and references therein). The lack of consistency between the results in the literature may be explained by the fact that the majority of the studies do not consider relevant biochemical and molecular aspects occurring in sperm or in the surrounding environment. However, we must also recognize that in these studies only the effect of a single EDC is generally evaluated. These single exposure studies are still the norm because it is still difficult to decipher the real impact of a particular contaminant if it is not tested alone. Such individual exposure assessment contrasts with the real word scenario in which we are permanently exposed to a myriad of EDCs, and thus such studies will misrepresent the real scenario. The accumulation of toxic substances in the human body has some clinical implications, especially in male fertility. Annually nearly 400,000 tonnes of pesticides are applied in the Europe [221], while in the United States the application of pesticides surpasses 450,000 tonnes, with the vast majority used in the agricultural sector [222]. Some studies have measured the levels of environmental contaminants in male reproductive fluids, with most of them dealing with metals (see systematic review by Sun and collaborators [223]). Other EDCs including pesticides [224], BPA [225], phtalates [226], perfluorooctane sulfonate (PFOS) and perfluorooctanoic acid (PFOA) [227] have also been detected in seminal male reproductive fluids including seminal plasma. After leaving seminiferous tubules, sperm undergoes several biochemical and morphological changes as they pass through the epididymis to become functionally competent, an intricate process that only ends after a series of several molecular events occurring within the female reproductive tract, called “sperm capacitation”. Sperm is transported against an increased hydrostatic pressure gradient from the seminiferous tubules to the cauda of epididymis [228]. Sex steroids mainly control this process, so it is expected that obesogens mimicking these hormones may disrupt it. Klinefelter and collaborators [229] reported alterations in sperm transit induced by environmental contaminants. These authors found that chloroethylmethanesulphonate, through the proximal segment of the epididymis, reduced T levels, accelerating sperm transit [229], eliciting androgen-dependent mechanisms. Evidence from castrated animals showed that T plays a crucial role in the sperm transport since a significant decrease of epididymal sperm storage [228,230] and an increased intraluminal pressure and contractility of the epididymis was found in these animals [231]. On the other hand, analogs of estrogens also modulate sperm transit, since Wisniewski and collaborators found that BPA administration decreased the sperm transit time through the caput and cauda of the epididymis. Yan and collaborators [232] also showed that TBT alters the secretory and absorptive activities of epididymal epithelium, so it may be expected that sperm transport is affected by organotins. As they pass through the epididymal duct, sperm motility gradually increases, as well as its fertilizing ability. This is attained due to rigorous crosstalk between sperm and epididymal epithelium resulting in several changes in the sperm plasma membrane [233]. The proteins secreted by the epithelium reaches the sperm membrane through apical blebs or small vesicles called epididymosomes, becoming a coating protein or an integral membrane protein. Alterations in the activity of epididymal proteins can also provide information concerning the maturation of sperm. This process is targeted by obesogens [234] as observed by Yan and collaborators [232], who found an abnormal morphology in sperm from TBT-treated rats which might have resulted in part from the decreased activity of epididymal acid phosphatase. TBT inhibits the activity of acid phosphatase [232], a lysosomal enzyme in the epididymal cells, involved in the lysosomal digestion and the removal of old and dead cells by phagocytosis [235]. Furthermore, acid phosphatase is also recruited for phosphorylation/dephosphorylation events associated with sperm maturation [236]. The acquisition of motility is one of the significant changes mediated by protein phosphorylation. Sperm motility is regulated by the phosphorylation profile in several parts of the flagellum and axoneme. Protein kinases and phosphatases coordinate this process, so obesogens that compromise the activity of these proteins affect motility. For instance, genistein is considered a natural obesogen and has been associated with male reproductive problems [237]. This isoflavonoid derived from soy is a competitive inhibitor of protein Tyrosine (Tyr) kinase, so it is expected that the exposure to this obesogen may hamper sperm maturation-related events, though the mechanism of action for genistein needs to be completely elucidated. Indeed, rat sperm incubated with genistein exhibited a decrease in both motility and morphology, with the most remarkable effects being observed for doses of genistein higher than 50 μg/mL and long-time exposure, illustrating the adverse effects of genistein on male fertility [237]. For lower doses (<50 μg/mL), no damages in the epididymal tissue and no significant alterations were observed in sperm parameters [238]. It is possible that high concentrations of genistein inhibit Tyr phosphorylation signaling. Recently, an association between the consumption of fruits and vegetables rich in pesticide residues and decreased sperm parameters was found [239]. The authors found that sperm count and sperm with normal morphology were reduced by 49% and 32%, respectively, when compared with men who had not been exposed to the same substances [239]. Another study also demonstrated that the conventional sperm parameters, specifically semen volume, concentration, morphology and viability of sperm, were altered in men chronically exposed to lead [240]. The majority of studies have focused on the adverse effects of EDCs on sperm quality (see Table 3), since this is the ultimate end-point for reproductive professionals and the one that defines male fertility. A meta-analysis by Wang and collaborators [241] has shown how classic EDCs compromise sperm quality, and although some of them were already banned from circulation, humans continue to be exposed and thus they continue to exert adverse effects. For instance, BPA has been consistently associated with changes in sperm parameters, especially in motility [242] and morphology [243]. Sperm from mice decreased in motility and all kinematic motion parameters after being exposed to 100 μM of BPA for six hours. The decreased sperm motility may be related to mitochondrial dysfunction since a concomitant decrease of ATP production was observed [244]. The majority of ATP required for sperm motility comes from mitochondrial respiration [245], therefore any condition/exposure leading to dysfunction of mitochondrial respiration will certainly compromise sperm motility [244]. Mitochondria are essential organelles for male fertility (see review [246]) and are localized in the sperm midpiece, forming a helicoidal sheath with the number of mitochondria and midpiece length varying with species [247]. Mitochondria in mature sperm have a condensed morphology with a compacted matrix, allowing for greater efficiency of energy production, but there are some species differences [248]. The relevance of mitochondria in sperm function was initially studied strictly concerning ATP production for motility. For instance, the activity of several complexes in the electron transfer chain of the inner mitochondrial membrane, as well as mitochondrial membrane potential (MMP), is correlated with sperm motility [249,250,251,252]. However, sperm mitochondria have unique features different than those of somatic cells, allowing them to efficiently drive important functions beyond sperm motility such as hyperactivation, capacitation, acrosome reaction, and fertilization [253,254]. Mitochondrial (dys)function has clinical relevance since it has been associated with male infertility [255,256]. Even changes in the mitochondrial genome have also been associated with male infertility due to compromised sperm quality, motility, and function [246]. Mitochondria are particularly sensitive to environmental exposures. It has been conjectured that the interaction between mitochondria and environmental exposure may be at the basis of several age-related diseases, such as Parkinson’s and Alzheimer’s diseases [257]. However, a detailed characterization of the “mitochondrial exposome” that allows us to understand the specific contribution of environmental chemicals in sperm mitochondrial dysfunction is necessary. Skibinska and collaborators exposed human sperm to different concentrations (10^−8^ and 10^−6^ M) of BPA and found alterations in the MMP [258]. In addition, these authors also found that human sperm incubated with E_2_, genistein, and BPA simultaneously decreased MMP and increased the production of superoxide anion. PCBs also induce sperm dysfunction since, under in vitro exposure to the mixture Aroclor 1254, a decreased motility, viability and acrosome reaction of rat sperm at all concentrations tested (10^−7^ M, 10^−8^ M, 10^−9^ M) was observed [259]. It was also found that Aroclor 1254 decreased the synthesis of ATP in a concentration-dependent manner, which elicits a decrease in the MMP. Additionally, in vitro exposure to Aroclor 1254 induced oxidative stress (OS), increased lipid peroxidation and induced mitochondrial cytochrome c release. The translocation of cytochrome c from mitochondria to the cytosol is the primary event in the mitochondrial pathway leading to the formation of apoptosomes and activation of the caspase cascade, which was confirmed by caspase-3 activation [259]. Although this has been observed in rat sperm, similar results were also reported in human sperm exposed to this pesticide [260]. Evidence from in vivo models has also corroborated in vitro findings (Table 3). Pant and collaborators [261] measured the levels of DEHP in the seminal fluid of men from rural (mean: 0.13–0.77 μg/mL) and urban areas and found negative associations with both sperm motility and mitochondrial integrity; however, the levels of DEHP were positively associated with sperm reactive oxygen species (ROS) production, lipid peroxidation, and nuclear DNA damages [261]. Obesogens have also been linked to developmental toxicity via DNA damage [262] and OS [263] (see Table 3). This is one of the most well-documented effects of environmental chemicals in male reproductive health [264,265]. OS may be part of the molecular basis of male infertility associated with environmental toxicants exposure. Chitra and collaborators [266] showed that administration of increasing doses of BPA is associated with a decreased activity of enzymes involved in the antioxidant system. Specifically, BPA decreased the activities of superoxide dismutase, catalase, glutathione reductase and glutathione peroxidase in the epididymal sperm of rats. This is of great relevance since an inefficient antioxidant system favors the production of ROS, as observed by the increased levels of hydrogen peroxide and consequent lipid peroxidation [266]. Recent data suggests that the anti-androgenic effects of DEHP may cause ROS production, lipid peroxidation and apoptosis of spermatocytes [267]. The production of these ROS seems to be related to Ca^2+^ mediated activation of the nicotinamide adenine dinucleotide phosphate complex, which may correlate with DEHP-induced Ca^2+^ entry [267]. In addition to the previous effects aforementioned, it should be further noted that obesogens also compromise the fertilizing ability of sperm. Even if the spermatozoa were not exposed to the effects of obesogens (both direct or indirect), they could always find a “hostile environment” in the female reproductive tract, since significant concentrations of PCBs in secretions and follicular fluid have been reported [268,269]. Thus, it is plausible that obesogens may, to some extent, compromise molecular mechanisms related to fertilization. For instance, it was reported that TBT decreased the activity of acrosin [232]. Acrosin is part of the mammalian sperm acrosome and is primarily found in an inactive form called proacrosin, which is only converted into the active form upon acrosome reaction. Acrosin activity is positively correlated with sperm morphology (regarding percentages of normal morphology) and negatively correlated with percentages of sperm with acrosome deficiency [270,271,272], so sperm with impaired acrosin activity will not be able to penetrate zona pellucida and fuse with the oocyte, thus contributing to male subfertility/infertility. Curiously, it has been shown that the administration of BPA to sperm leads to a dose-dependent induction of acrosome reaction, suggesting that BPA, especially at high concentrations, modulates Tyr phosphorylation by regulating PKA activity. A significant inhibitory effect on fertilization and early embryonic development was also found, which might be due to decreased sperm motility and early acrosome reaction [244]. Rahman and collaborators [244] proposed that decreased sperm motility and early acrosome reaction might result in part of the downregulation of sperm’s actin. Actin is a cytoskeletal protein localized in the acrosome, equatorial region, and tail [273]. The localization of actin in the tail illustrates its function in sperm motility, while actin in the sperm head may be involved in acrosome reaction [273]. Sperm functionality is reflected by the ability to fertilize oocytes, where sperm motility, membrane integrity, and even mitochondrial activity are crucial to allow the interaction between sperm and the female gamete [274,275,276]. Overall, there is a need for further evaluation concerning the properties of these contaminants and their reproductive toxicity to establish safety thresholds. This might be important to confirm which of environmental pollutants are contributing to the current decline of male fertility.

## 11. Conclusions

There is an intense debate concerning the contribution of environmental cues to male fertility. Several studies concerning this topic reveal a negative impact of external compounds in the reproductive function of individuals exposed from very early periods (even in prenatal), not only through indirect effects mediated by changes in hormonal levels and the HPT axis, but also by direct changes in the reproductive tissues. The toxic effects may compromise spermatogenesis and end up in the impairment of the molecular composition of sperm quality and function. Definitive conclusions on how obesogens affect the cellular and molecular mechanisms involved in the production and function of sperm are, however, hard to establish. First, when exposure occurs, it is often through a combined mixture of environmental chemicals and not due to a single compound. Besides, sometimes there is also a long latency period between the exposure and the noticed effects, which further hampers the association to a specific agent. However, from a clinical point of view, it is imperative to identify mechanisms by which testicular metabolic pathways are compromised after exposure to obesogens. SCs have arisen as one of the best in vitro and/or ex vivo models that have allowed researchers from this area to understand the molecular basis by which obesogens impact male fertility. In addition, the use of experimental animal models must be applied to permit detailed knowledge on the effects of obesogens on testicular cell-specific functions. Apart from the elucidation of all those points, it is needed to unveil why and how the metabolic alterations caused by what male individuals are exposed to may result in molecular changes that are carried out by male gametes and thus may be passed to the next generations. In fact, in this work we gathered several studies showing the relationship between the exposure to environmental contaminants and epigenetics, and identified several toxicants that modify epigenetic marks, particularly regarding DNA methylation. This field reveals a considerable potential for precocious disease diagnosis, since future research may allow prediction of which contaminants would put exposed individuals at risk, which individuals will be more susceptible to develop a disease, and whether such epigenetic alterations increase the risk of illness. Since we are permanently exposed to environmental contaminants that may induce a myriad of effects that persist through the generations, this topic will undoubtedly become a matter of debate for professionals of the environmental toxicology and reproduction fields.

## Figures and Tables

**Table 1:** Summary of proposed obesogens and possible sources of exposure.

Obesogens	Main Sources	References
2,4-D	Herbicides	[87]
B[α]P	Residential wood burnings, cigarette smoke, charbroiled food, coal tar, and automobile fume emissions	[88]
BPA	Food and drink packaging plastics, medical devices, and thermal paper	[89]
Chlorpyrifos	Insecticides	[90]
Diazinon	Insecticides	[91]
Diethylstilbestrol	Cattle feed and medical treatments for breast and prostate cancers	[92]
Fructose	Fruit, vegetables, and honey	[93]
Genistein	Soybeans and soy products, fava beans, and coffee	[94]
Lead	Diet, dust, ceramics, paints, and infant toys	[95]
MSG	Food additives and natural foods such as tomatoes and cheese	[96]
Nicotine	Tobacco, insecticides, and nightshade plants	[97]
Parabens	Preservatives in personal care products	[98]
Parathion	Insecticides and acaricides	[99]
PBDEs	Flame retardant in building materials, electronics, furnishings, plasticizers, and textiles	[100]
PCBs	Electric equipment, transistors, plasticizers, surface coatings, paints, and carbonless copy paper	[101]
PFOA	Crawl and stain repellent on carpets, furniture, waterproof clothing, mattresses, and microwavable food items, non-stick kitchen utensils	[102]
Phthalates	Plastics, PVC products, infant toys, detergents, and personal care products	[103]
TBT	Antifouling paints, plastic products, silicones, and polyurethanes	[6]
TZD	Antidiabetic drugs	[104]

Legend: 2,4-D: 2,4-dichlorophenoxyacetic; B[α]P: benzo[α]pyrene; BPA: bisphenol A; MSG: monosodium glutamate; PBDEs: polybrominated diphenyl ethers; PCBs: polychlorinated biphenyls; PFOA: perfluorooctanoic acid; PVC: polyvinyl chloride; TBT: tributyltin; TZD: thiazolidinediones.

**Table 2:** Summary of the main obesogens and their proposed effects in both glucose and lipid metabolism, and their toxic effects in reproductive cells/tissues of mammals.

Obesogens	Specie(s)/ Tissue(s)/Cells		Doses	Glycolytic Metabolism Effects	Lipid Metabolism Effects	Toxic Effects
2,4-D	Rat SCs		100 nM, 10 µM, 1 mM	↓GLUT3, PFK1 LDH mRNA, ↓Lactate production [122]	n.d.	n.d.
BPA	Rat testis		0.005, 0.5, 50, 500 µg/Kg body wg/day	↓IRS-1, ↓GLUT2 [123] ↓HEX, ↓PFK [125]	n.d.	n.d.
CPYF	Rat testis		0, 2.7, 5.4, 12.8 mg/Kg body wg	↑LDH [126]	n.d.	n.d.
Lead	Rat SCs		0.01, 0.05, 0.1 mM	↑Lactate production [127]	n.d.	↑Lipid peroxidation, ↑CAT activity, ↑GSH, ↓SOD activity [127]
PCBs	Rat SCs		10^−7^ M (PCB22) 10^−8^ M (PCB77)	↑Lactate production [128]	n.d.	n.d.
PIO	Rat SCs		1, 10, 100 µM	↑Glucose uptake ↓GLUT3 ↑Lactate production ↑LDH ↑MCT4[104]	n.d.	n.d.
PTLs	Rat	Testis	CE-2 diet with 2%(mass) of DEHP	n.d.	↓ACC ↑LCAD ↑3KACT [129]	n.d.
		SCs	0.1–200 µM	↑Pyruvate production ↑Lactate production [124]	n.d.	n.d.
TBT	Rat	Testis	10, 20, 30 mg/Kg of body wg	n.d.	n.d.	↓BTB ↑TBARS ↑ROS ↓Steroidogenesis [130]
		LCs	300–3000 nM	n.d.	n.d.	↓MMP ↓Steroidogenesis ↑Apoptosis [131]
		SCs	0.1 nM, 10 nM	↓Glucose uptake ↓Pyruvate uptake ↓GLUT1 ↓Lactate production [132]	n.d.	n.d.
		SCs/GCs co-culture	300, 600 and 1000 nM	n.d.	n.d.	↑Apoptosis [133]

Legend: 2,4-D: 2,4-dichlorophenoxyacetic; 3KACT: 3-ketoacyl-CoA thiolase; ACC: acetyl CoA carboxylase; BPA: bisphenol A; BTB: blood–testis barrier; CAT: catalase; CPYF: chlorpyrifos; DEHP: bis(2-ethylhexyl) phthalate; GCs: germ cells; GLUT1: glucose transporter 1; GLUT2: glucose transporter 2; GLUT3: glucose transporter 3; GSH: glutathione; HEX: hexokinase; IRS-1: insulin receptor substrate 1; LCAD: long-chain acyl-CoA dehydrogenase; LCs: Leydig cells; LDH: lactate dehydrogenase; MDA: malondialdehyde; MMP: mitochondrial membrane potential; n.d.: non-determined; PCBs: polychlorinated biphenyls; PFK: phosphofructokinase; PFK1: phosphofructokinase 1; PIO: pioglitazone; PTLs: phthalates; SCs: Sertoli cells; SOD: superoxide dismutase; T: testosterone; TBARS: thiobarbituric acid reactive species; TBT: tributyltin; ROS: reactive oxygen species; wg: weight.

**Table 3:** Summary of the main studies focusing on the effects of obesogens on sperm quality and sperm parameters.

Sperm Parameters	Species	Obesogens	Doses/Concentrations	Outcomes	References
Motility	Human	BPA	n.a.	↓	[277,278]
	Rat		0.0001, 0.01, 1, and 100 mM	↓	[279]
	Human	PTLs	n.a.	↓	[261,280,281]
	Human	POPs	n.a.	↓	[282]
	Human	PCBs	n.a.	↓	[283,284,285]
	Rat	Aroclor 1254	10^−9^, 10^−8^, and 10^−7^ M	↓	[259]
	Human	CB153	n.a.	↓	[286,287]
	Human	DDT	n.a.	↓	[287,288,289]
	Human	p,p’-DDE	n.a.	↓	[97]
			53.89, 269.45, and 538.9 mg/L	↓	[290]
			10, 25, 50, and 100 mM	↓	[291]
	Human	Alachlor	0.18, 0.37, 0.90, and 1.85 mM	↓	[260]
Concentration	Human	BPA	n.a.	↓	[278]
	Human	PTLs	n.a.	↓	[261,281,292]
	Human	PFOS, PFOA	n.a.	↓	[293,294]
	Human	PCBs	n.a.	↓	[283,295]
	Human	Pesticides	n.a.	↓	[296]
	Human	DDT	n.a.	↓	[288]
Morphology	Human	BPA	n.a.	↓	[278]
	Human	PTLs	n.a.	↓	[261,297]
	Human	PCBs	n.a.	↓	[283,284]
	Human	DDT	n.a.	↓	[288,289]
	Human	p,p’-DDE	n.a.	↓	[289]
Viability	Human	BPA	n.a.	↓	[277,278]
	Human	Alachlor	0.18, 0.37, 0.90, and 1.85 mM	↓	[260]
		p,p’-DDE	1, 10, 25, 50, and 100 µM	↓	[291,298]
		PTLs	5.73, 28.65, and 57.3 mg/mL	↓	[299]
Mitochondrial function	Human		0.18, 0.37, 0.90, and 1.85 mM	↓	[260]
		p,p’-DDE	1, 10, 25, 50, and 100 µM	↓	[291,298]
	Rat	Aroclor 1254	10^−9^, 10^−8^, and 10^−7^ M	↓	[259]
Oxidative stress	Human	Alachlor	0.37 and 1.85 mM	↑	[260]
	Human	B[α]P	500 µM	↑	[300]
	Rat	Aroclor 1254	10^−9^, 10^−8^, and 10^−7^ M	↑	[259]
Lipid peroxidation	Rat	Aroclor 1254	10^−9^, 10^−8^, and 10^−7^ M	↑	[259]
Capacitation	Human	p,p’-DDE	25, 50, and 100 µM	↓	[291]
	Mouse	BPA	0.0001, 0.01, 1, and 100 µM	↓	[279]
	Human	B[α]P	12.5, 25, 50, and 100 µg/mL	↑	[301]
	Human	Genistein	1, 10, and 100 nM	↑	[302]
	Boar		0.001, 0.01, 0.1, 1, 10, and 100 µM	↑	[303]
	Mouse		1, 10, 100, and 1000 nM	↑	[304]
Acrosome integrity	Human	p,p’-DDE	1, 10, 25, and 50 µM	↓	[298]
	Rat	Aroclor 1254	10^−9^, 10^−8^, and 10^−7^ M	↑	[259]
	Human	B[α]P	12.5, 25, 50, and 100 µg/mL	↓	[301]
	Mouse	BPA	0.0001, 0.01, 1, and 100 µM	↓	[279]
	Human	Genistein	1, 10, and 100 nM	↑	[302]
	Boar		0.001, 0.01, 0.1, 1, 10, and 100 µM	↑	[303]
	Mouse		1, 10, 100, and 1000 nM	↑	[304]
Sperm-oocyte interaction	Mouse	BPA	0.0001, 0.01, 1, and 100 µM	↓	[279]
	Rat	PCB77	0.01, 0.1, 1, and 10 µg/mL	↓	[305]
	Mouse	Genistein	100 nM	↑	[304]

Legend: B[α]P: benzo[α]pyrene; BPA: bisphenol A; DDT: dichlorodiphenyltrichloroethane; p,p’-DDE: p,p’-dichlorodiphenyldichloroethylene; PCBs: polychlorinated biphenyls; PFOS: perfluorooctanesulfonic acid; PFOA: perfluorooctanoic acid; POPs: persistent organic pollutants; PTLs: phthalates; Ref.: references.
